# The significant role of redox system in myeloid leukemia: from pathogenesis to therapeutic applications

**DOI:** 10.1186/s40364-020-00242-z

**Published:** 2020-11-11

**Authors:** Natasha Mupeta Kaweme, Shu Zhou, Geoffrey Joseph Changwe, Fuling Zhou

**Affiliations:** 1 Department of Hematology, Zhongnan Hospital affiliated to Wuhan University, No. 169 Donghu road, 430071 Wuhan, P.R. China; 2 School of Medicine, Shandong University, No. 44, Wenhua West Road, Jinan, 250012 P.R. China

**Keywords:** Acute myeloid leukemia, Reactive oxygen species, Oxidative stress, Oncogene mutations, Jab1/COPS5, Antioxidants

## Abstract

**Background:**

Excessive generation of reactive oxygen species (ROS) in the presence of a defective antioxidant system can induce cellular damage and disrupt normal physiological functions. Several studies have revealed the unfavorable role of ROS in promoting the growth, proliferation, migration, and survival of leukemia cells. In this review study, we summarize the mechanisms of ROS production and its role in leukemogenesis, counteractive effects of antioxidants, and implicate the current ROS-dependent anticancer therapies in acute myeloid leukemia.

**Body:**

The dysregulation of the redox system is known to play a significant role in the pathogenesis of leukemia. Leukemia cells generate high levels of ROS, which further increases the levels through extra pathways, including mitochondrial deoxyribonucleic mutation, leukemic oncogene activation, increased nicotinamide adenine phosphate hydrogen (NADPH), and cytochrome P450 activities. Aforementioned pathways once activated have shown to promote genomic instability, induce drug resistance to leukemia medical therapy, disease relapse and reduce survival period. The current standard of treatment with chemotherapy employs the pro-oxidant approach to induce apoptosis and promote tumor regression. However, this approach retains several deleterious effects on the subject resulting in degradation of the quality of life. Nevertheless, the addition of an antioxidant as an adjuvant drug to chemotherapy alleviates treatment-related toxicity, increases chemotherapeutic efficacy, and improves survival rates of a patient.

**Conclusion:**

Acute myeloid leukemia remains a daunting challenge to clinicians. The desire to achieve the maximum benefit of chemotherapy but also improve patient outcomes is investigated. ROS generated through several pathways promotes leukemogenesis, drug resistance, and disease relapse. Chemotherapy, the mainstay of treatment, further upregulates ROS levels. Therefore, the addition of an antioxidant to leukemia medical therapy alleviates toxicity and improves patient outcomes.

## Introduction

Reactive Oxygen Species (ROS) is a variety of chemically-active and oxygen-containing molecules that are naturally produced during cellular metabolism [1] and are involved in regulating normal biological cell functions, cell signaling, and homeostasis [2, 3]. They play critical roles in promoting health and longevity, and antimicrobial phagocytosis by cells of the innate immune system [4]. ROS in low concentrations is beneficial to supporting cell proliferation and continuity of pathways, whereas high concentration leads to the damage of deoxyribonucleic acid (DNA), proteins and lipids in normal or precancerous cells and promotes malignancy transformation. Therefore, the dynamic regulation of ROS in the redox microenvironment is critical to cell survival and function.

Typically, an intracellular balance between the generation and elimination of ROS is maintained by antioxidants or specific regulatory pathways, with the major players being Glutathione and Thioredoxin. In hematological malignancies distinctly, ROS perform a double role in tumorigenicity [5]. At high levels, ROS suppress tumor growth and induce apoptosis, which is the primary mode of action of chemotherapeutic agents [6]. In contrast, at low levels, ROS protect the cell from apoptosis and promote cell survival, growth, proliferation, migration, and drug resistance.

A pervasive condition in which an imbalance between ROS production and the response of endogenous antioxidant defense systems resulting in ROS accumulation is known as oxidative stress (OS) [7]. OS is implicated in several acute and chronic diseases, including hematological malignancies such as acute myeloid leukemia (AML), chronic myeloid leukemia (CML), acute lymphoblastic leukemia (ALL), and myelodysplastic syndromes. AML is a highly aggressive hematopoietic malignancy characterized by highly proliferative blast cells. It is the most common acute leukemia in adults with an increasing incidence with age and high relapse rates [8]. Despite current advancement in the treatment of AML, refractory disease remains prevalent, with disease relapse being the major cause of treatment failure [9]. The current AML management guidelines largely rely on high-dose chemotherapy with cytarabine- and anthracycline-based regimes, and allogeneic hematopoietic stem cell transplant (HSCT) [10].

The use of intensive chemotherapy leads to increased ROS generation and ROS-induced cytotoxicity. Elevated ROS levels and other molecular mechanisms of ROS production, including nicotinamide adenine dinucleotide phosphate (NADPH) oxidase, mitochondrial electron transport chain (mtETC), leukemic oncogene activity, xanthine oxidase, and cytochrome P450, are the key feature of leukemia cells [11]. AML is associated with an inadequate antioxidant status resulting in an imbalance in the redox microenvironment leading to OS. Antioxidants control the OS state and provide a major defense line in blocking the harmful effects of ROS [12]. Several studies support the application of antioxidants in leukemia treatment to improve patient outcomes. This review study aims at summarizing the mechanisms of ROS production and its role in leukemogenesis, counteractive effects of antioxidants and implicate the current ROS-dependent anticancer therapies in AML.

## ROS and the mechanisms of ROS production

ROS are a heterogenous group of small molecules and free radicles, which include hydrogen peroxide, superoxide anions, ozone, singlet oxygen, organic peroxides and hypochlorous acids [13]. In hematopoietic stem cells (HSCs), ROS are generated in the mitochondria through NADPH oxidases and other ROS-related metabolic pathways such as polyamine metabolism, cytochrome P450, and xanthine oxidase [14]. Overproduction of ROS without adequate counteraction by antioxidants results in OS, which mediates the damage of cell structure and membranes, DNA, lipids, and proteins [15]. In hematological malignancies, OS is generated from chemotherapy and radiotherapy use. The effect of OS on cells can be acute or chronic. OS’ chronic effect is associated with little oxidative damage, accumulating over time in the life cycle of a cell, eventually interrupting normal cellular function and promoting cancerous changes. Acute OS is implicated in acute illness such as sepsis, cardiovascular accidents, myocardial infarction and is a useful measurement in evaluating acute illness [16].

ROS source can be either endogenous or exogenous. Cellular metabolism gives rise to endogenous ROS through mitochondria-catalyzed transport reactions, cytochrome P450 metabolism, and inflammatory activities involving neutrophils, eosinophils, peroxisomes, and macrophages [17]. Mitochondria are the primary source of free radicals in living organisms, generating an estimated 2–3 nmol of superoxide/min per mg of protein. Evidence shows that complex III of the mitochondria is the main site of ROS production in normal metabolism. Cytochrome P450 enzyme induces the production of superoxide anion and hydrogen peroxide after the breakdown of the P450 catalytic cycle [18]. Macrophage activation during inflammation may increase oxygen uptake, leading to increased superoxide anion, nitric oxide, and hydrogen peroxide [19]. The conversion of hypoxanthine to xanthine and its subsequent transformation into uric acid is catalyzed by xanthine oxidase with superoxide anion as a by-product [20].

Exogenous sources include cigarette smoke, which naturally contains free radicals and organic compounds like superoxide and nitric oxide, which activate endogenous mechanisms and lead to an accumulation of neutrophils and macrophages, worsening oxidant injury [21]. Exposure to ozone may cause lipid peroxidation and induce airway invasion by neutrophils and compromise lung function even in healthy individuals. Hyperoxia is a condition of high oxygen levels than the average partial pressure of oxygen in the lungs or other tissues, results in increased production of reactive oxygen and nitrogen species [22]. Exposure to radiation, xenobiotics, barbiturates, chlorinated compounds, air and water pollution, alcohol, and heavy metals are implicated in ROS production [23].

## Oxidative stress-induced cellular injury

OS, as defined, occurs when intracellular antioxidants are unable to counteract the pro-oxidants, causing damage to various cell components and triggering activation of specific pathways [24]. The three main pathways by which OS causes cellular injury are lipid peroxidation of membranes, oxidative modification of proteins, and DNA damage [25]. In lipid peroxidation, damage to the cell membrane and other lipid-containing structures gives rise to primary products known as lipid hydroperoxides which alter membrane structure and endanger the cell [26]. The secondary products of lipid peroxidation, aldehydes, have a ‘no-charge’ property which allow them easily to permeate through the membranes into cytosol and cause extensive damage both inside and outside the cell. Amongst the aldehydes, malondialdehyde is highly mutagenic, and 4-hydroxynonenal is highly toxic [27].

Oxidative modification of proteins results in the formation of protein carbonyl derivatives which together with advanced oxidation protein products, advanced glycation end products, and S-nitrosylated proteins serve as markers for ROS-mediated protein damage [28]. The secondary products of lipid peroxidation, malondialdehyde and 4-hydroxynonenal, react with and modify proteins at several amino acid side chains leading to a formation of stable adducts and a change in the function and fate of that protein [25, 29]. The aldehyde protein-adducts play a vital role in physiological processes but are associated with disease pathogenesis, and influence the aging process and cellular senescence [30].

Studies have shown that ROS and lipid peroxidation products affect genomic and mitochondrial DNA (mtDNA), inducing DNA damage to nuclear and mitochondrial compartments. Furthermore, the replication of damaged DNA before repair leads to DNA mutations and genomic instability [31]. DNA double-strand breaks are the most destructive impairment compared to single-strand breaks. They cause severe genetic mutations leading to oncogene activation or tumor suppressor gene inactivation, gene expression modification and tumor development [32]. Though not harmful, single-strand breaks can result in serious lesions if not repaired early [33]. 8-oxoGuanine (8-OHG), an oxidation product generated in DNA through deoxyguanosine (dG) oxidation, and its nucleoside form 8-oxo-2′-deoxyguanosine (8-OHdG), are useful markers of oxidative DNA damage both in vivo and in vitro [34]*.* The formation of 8-OHG/8-OHdG, DNA lesions is responsible for mutagenesis and carcinogenesis [35, 36]. Products of lipid, protein, and DNA damage have been investigated and extensively studied as biomarkers of OS in leukemia by earlier pioneers [28, 37–39], as illustrated in (Table 1**)**.

**Table 1:** Extensively studied biomarkers of oxidative stress in patients with leukemia

Biomarkers	Clinical Significance	Pioneer(S)
MDA,TAC	To determine the involvement of OS in AML development and implicate biomarkers of OS in disease diagnosis and prognosis	Tsamesidis et al. 2019
PC,TBARS,LOOH	To evaluate and quantify products of protein carbonylation and lipid peroxidation as valuable indicators for OS and disease progression in CML	Singh et al. 2009
MDA	To estimate MDA concentration as a marker of OS in AML patients receiving treatment	A. Hlavackova et al. 2019
TBARS, PC	To determine oxidative damage from increased OS and decreased antioxidant defense in ALL patients	Battisti et al. 2008

Abbreviations: *OS* Oxidative stress, *MDA* Malondialdehyde, *TAC* Total antioxidant capacity, *PC* Protein carbonyl, *TBARS* Thiobarbituric acid reactive substances, *LOOH* Lipid hydroperoxide, *AML* Acute myeloid leukemia, *CML* Chronic myeloid leukemia, *ALL* Acute lymphoblastic leukemia

## ROS in myeloid leukemia

Leukemogenesis is a multistep process caused by mutations in DNA repair genes, oncogenes, and tumor suppressor genes. Thus, it is difficult to identify a single driving force as to the cause of leukemogenesis. ROS are implicated in the pathogenesis of hematological malignancies [40, 41]. Increased ROS production remains a key feature of human tumor cells, of which leukemic cells are no exception. In leukemia, an increase in ROS and antioxidants, as an adaptive protective defense, is indicative of OS, resulting in genetic mutations, chromosomal alternations and contributes to carcinogenesis [42]. It has long been known that ROS have a crucial role in regulating the balance between self-renewal and myeloid differentiation of HSCs. Generally, ROS exist at low levels in HSCs to maintain pluripotency. Several theories support the mechanisms that promote leukemogenesis.

### Primary ROS production in leukemia

NADPH oxidases (NOX) are an important primary source of ROS in leukemia. Studies have shown that over 60% of primary AML blasts produce high levels of NOX-derived ROS, promoting cell proliferation and survival in AML [41]. The increased ROS levels are associated with reduced GSH levels and depletion of antioxidant defense proteins, mtDNA and peroxiredoxin [43], demonstrating that NOX-derived ROS develop adaptative mechanisms to suppress stress signaling that would normally limit this response. The mtETC complex is another major source of endogenous ROS in any known cell type, and through oxidative phosphorylation, the mitochondria provide adenosine triphosphate. Mutations in mtDNA in leukemia cells lead to alternations in the mtETC, enhancing ROS production. In chronic lymphocytic leukemia (CLL), mitochondrial ROS are implicated in promoting genetic instability and drug resistance [44].

Leukemic blasts continuously generate excessive ROS involved in the regulation of intracellular signaling pathways and modulation of cells in the microenvironment, thereby promoting leukemogenesis [45]. In the leukemic bone marrow microenvironment, NADPH oxidase-2 derived ROS drive mitochondrial transfer between leukemic cells and bone marrow mesenchymal stromal cells through tunneling nanotubules enhancing metabolic activity in AML cells [46]. In addition to leukemic cells, immune cells such as neutrophils, eosinophils, mononuclear phagocytes, tumor-associated macrophages, regulatory T cells and myeloid-derived suppressor cells are implicated in ROS production in the microenvironment, facilitating tumor growth [47].

### Metabolic pathways of ROS production

Xanthine oxidase/dehydrogenase has been implicated in several diseases, though its involvement in leukemia has not been adequately researched. It has been reported that increased xanthine oxidase activity and uric acid production is induced in myeloid leukemia cells. Activator protein-1 and hypoxia-inducible factor 1 transcription complexes are implicated in ligand-induced xanthine oxidase upregulation [48]. In relapsed AML, elevations in xanthine oxidase activity are found, contributing to increased ROS levels [49]. Cyclooxygenases and lipoxygenases, enzymes produced by tumor cells, are proved to promote tumorigenesis by directly promoting and enhancing cell proliferation, migration, and survival. The expression of cyclooxygenase-2 is highly elevated in CML and CLL and is associated with poor prognosis and enhancement of survival and proliferation of malignant cells [50].

Genetic polymorphisms of cytochrome P450 promote ROS generation in leukemia, though more investigation is required to clarify this theory. The bone marrow microenvironment is demonstrated to contribute to drug resistance, and this is associated with the expression of cytochrome P450 enzymes. In AML, fms-like receptor tyrosine kinase 3 (FLT3) mutations are among the most common genetic alterations. The introduction of FLT3 tyrosine kinase inhibitors (TKIs) to target AMLs with mutant FLT3 yields promising results. Still, it fails to achieve durable responses due to minimal residual disease resulting in disease relapse associated with resistance to FLT3 TKIs [51]. Cytochrome P450 enzymes contribute to bone marrow-mediated FLT3-AML protection from FLT3 inhibitors [52].

### Oncogene activity-induced ROS production

Alternations in leukemic ‘driver’ oncogenes caused by chromosomal translocations and mutations are found in all forms of leukemia. Breakpoint cluster region-Abelson (BCR-ABL) mutation caused by a chromosomal translocation between chromosomes 9 and 22 is commonly found in CML and a subtype of ALL. It regulates proliferation and survival signaling in leukemia. Studies have shown that BCR-ABL expression can induce elevated ROS production, contributing to genomic instability and progression to blast-crisis in CML [53]. Mutations such as M244I, E255K, and T315I, caused by BCR-ABL induced ROS within the BCR-ABL kinase domain itself, promote resistance to BCR-ABL TKIs [54]. The idea that BCR-ABL oncogene induces ROS production responsible for the aberrant proliferation, cellular signaling, and resistance to TKIs can be validated.

FLT3-ITD (fms-like receptor tyrosine kinase 3-internal tandem duplication), Ras, and c-Kit are driver leukemic-oncogenes activated by mutations. FLT3-ITD is the most prevalent mutation in acute leukemia and is expressed in about 30% of AML patients [55]. FLT3-ITD mutation induces ROS elevation through NADPH oxidases, and its constitutive activity promotes proliferation and survival signaling in AML cells [56]. Ras mutations, found prevalent in about 20% of AML cases cause cell transformation through NADPH oxidase driven ROS, thereby promoting survival and growth factor-independent proliferation in human CD34+ cells [57]. C-Kit, much like Ras, has a role in ROS production by regulating NOX activity [58]. B-cell lymphoma 2 (Bcl-2) functions as an oncogene in AML, ALL, and CLL. Though previous studies demonstrated the inhibition potential of Bcl-2 by inducing antioxidant proteins in cancer cells [59], recent research has shown that Bcl-2 can promote ROS production in human leukemia cell lines, particularly. Bcl-2 family proteins regulate cancer cell migration, invasion, and metastases [60].

Recently, mutations in the Isocitrate dehydrogenase 1 and 2 (IDH1 and IDH2) genes have been reported common in AML, myelodysplastic syndromes, and angioimmunoblastic T-cell lymphomas [61]. When a mutation occurs, IDH1 and IDH2 convert α-ketoglutarate (α-KG) to 2-hydroxyglutarate (2-HG) while consuming NADPH instead of their normal function of converting isocitrate to α-KG. 2-HG accumulates to high levels in cancer cells inhibiting α-ketoglutarate-dependent dioxygenase enzymes, thereby expressing mutant IDH1 or IDH2 [62]. IDH mutations alter redox homeostasis by diminishing cellular NADPH, and through 2-HG, which acts as an ‘oncometabolite’ and contributes to leukemia transformation and progression [63].

In summary, mtETC and NOX complex are major sources of ROS in leukemic cells. Alternations in leukemic oncogene activity control several NOX components and induce elevated NADPH levels, resulting in amplified ROS production. The metabolic/detoxification enzymes, xanthine oxidase/dehydrogenase and cytochrome P450 also contribute to ROS production in leukemia cells. The mentioned pathways are implicated in leukemogenesis and leukemia progression, either by promoting genomic instability, cell proliferation, and survival, or creating drug resistance. Understanding the role of each ROS-producing pathway in leukemogenesis makes them potential targets for research in AML treatment.

## Oncogene Jab1/COPS5 in myeloid leukemia

Recent research demonstrates the role of Jab1/COPS5 overexpression in tumorigenesis, signaling pathways and the development of oncogene inhibitors [64]. C-Jun activation domain-binding protein-1 (Jab1), which was initially identified as a c-Jun coactivator, is known to modulate cell proliferation, cell cycle, and apoptosis. Additionally, it regulates genomic instability, DNA damage response, and affects intracellular signaling through its existence as a member of the COP9 signalosome complex fifth subunit (COPS5, CSN5) [64, 65]. As a multifunctional complex, dysregulation of Jab1/COPS5 deactivates several tumor suppressors, and activates oncogenes, promoting oncogenesis. Furthermore, Jab1 stabilizes hypoxia-inducible factor-1alpha and c-Jun and acts as a transcription co-factor for MYC, regulating transcriptional activation of genes involved in cell proliferation, angiogenesis, and invasion [66]. As an oncogene, Jab1/COPS5 is aberrantly overexpressed in several human cancers and implicated in facilitating carcinogenesis.

In cancer, dysregulation of Jab1/COPS5 expression may originate from three regulatory mechanisms: gene amplification, microRNAs, and other signaling transduction pathways such as IL6-Stat3 (signal transducer and activator of transcription 3) signaling, HER2-AKT (human epidermal growth factor receptor 2-protein kinase B) signaling, and BCR-ABL signaling. However, more investigation is required to validate them [67]. A recent study was undertaken to investigate the role of ROS and OS on gene expression in the development and relapse of acute monocytic leukemia. In this study, blood samples obtained from AML patients at initial diagnosis were compared with AML cells from the same patients obtained at relapse. Collective data demonstrated that at both diagnosis and disease relapse, increased ROS production and low capacity of antioxidant enzymes were characteristics of AML-M5. High gene expression levels of Jab1 and Trx were associated with disease progression and poor prognosis in relapsed AML-M5, showing that increased levels of ROS stimulate aberrant gene expression and promote the proliferation of leukemic blasts. Jab1 is a new target in the ROS pathway and plays a critical role in the pathogenesis of AML-M5 through its interaction and positive regulation of Trx expression [68].

**Fig. 1:**
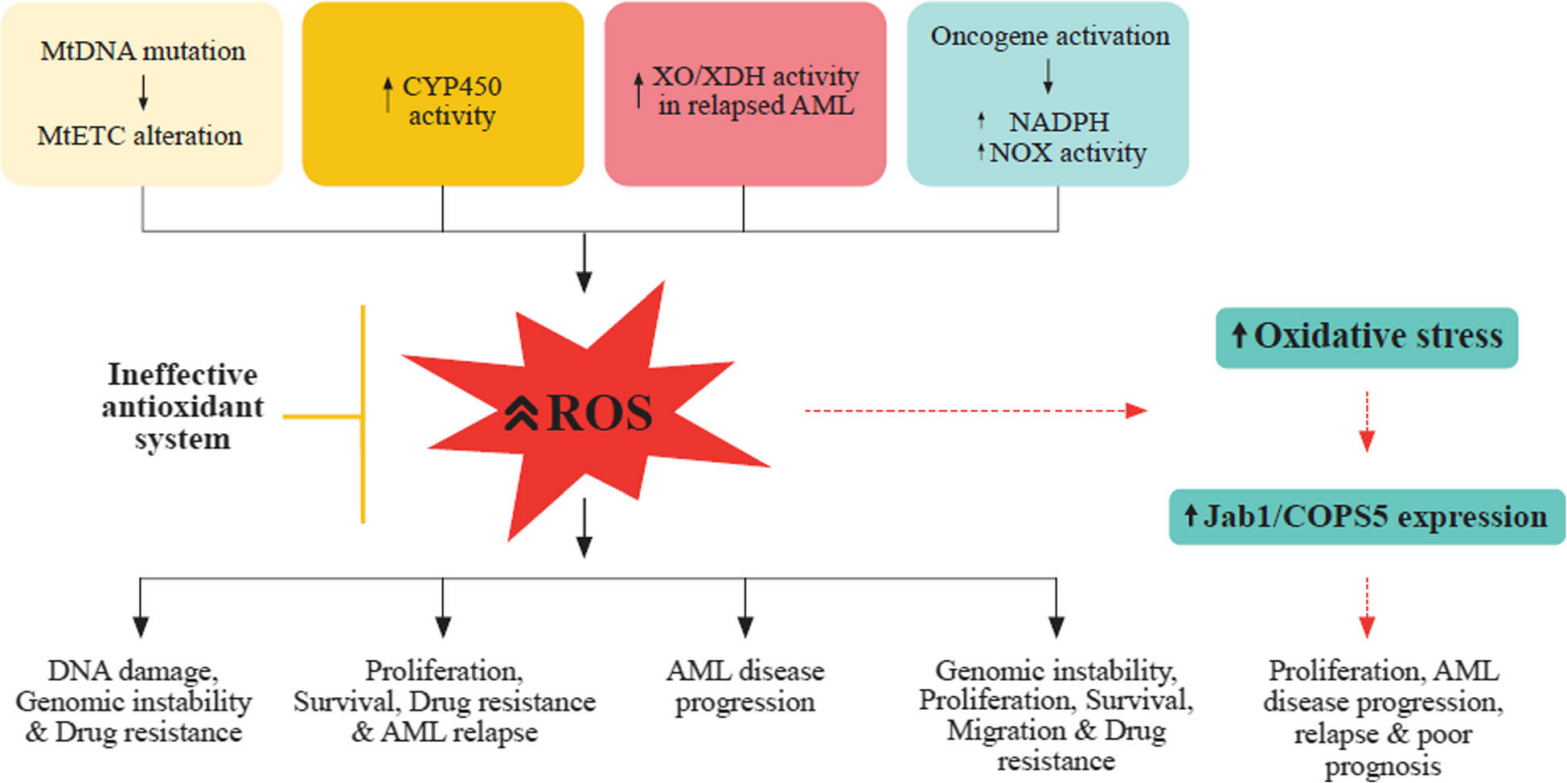
The different mechanisms of ROS generation in leukemia cells and their role in leukemogenesis. **(1)** MtDNA mutations lead to alternations in the mtETC, resulting in increased ROS production promoting DNA damage, genomic instability, and drug resistance in AML. **(2)** Activated genetic polymorphisms of CYP450 in AML, ALL, and CLL, generate high ROS levels contributing to cell proliferation, survival, drug resistance, and disease relapse. **(3)** Elevated XO/XDH activity leads to increased ROS & OS attributed to AML relapse and progression. **(4)** Leukemic oncogene activity (BCR/ABL, Flt3-ITD, Ras, c-Kit, JAK2 V617F) induce NADPH elevation and increased NOX activity resulting in elevated ROS levels which promote DNA damage, genetic instability, proliferation, survival, migration, and drug resistance in leukemic cells. **(5)** Increased ROS generated from leukemic cells, oncogene mutations, abnormal metabolism with an ineffective antioxidant system results in increased OS which aberrantly expresses Jab1/COPS5 leading to AML proliferation, progression, and relapse. **Abbreviations**: mitochondrial ETC, electron transport chain; CYP450, cytochrome P450; XO, xanthine oxidase; XDH, xanthine dehydrogenase; AML, acute myeloid leukemia; BCR/ABL, Breakpoint cluster region-Abelson leukemia virus; Flt3-FTD, FMS-like tyrosine kinase 3-internal tandem duplication; c-Kit, receptor tyrosine kinase; JAK2 V617F, Janus kinase 2 V617F; Jab1/COPS5, c-Jun activation domain-binding protein-1

A summary of the different ROS generation mechanisms in leukemia cells and their role in leukemogenesis are shown **(**Fig. 1**).**

## Cellular antioxidants in myeloid leukemia

Antioxidants can either be upregulated or downregulated in leukemia cells. The upregulation of antioxidants is observed in acute leukemia, in which expression of superoxide dismutase (SOD), GSH, Trx, Peroxiredoxin is increased. Dysregulation of antioxidants alters redox homeostasis and may have beneficial or detrimental effects on leukemic cells. Upregulation of antioxidant activity increases the resistance of leukemic cells to deleterious ROS effects. Downregulation of antioxidant activity has been reported in lymphocytes from ALL [69] and CLL [70] patients in which both SOD and catalase (CAT) activity was reduced.

Heme oxygenase (HO) has defense properties against ROS in leukemia and is implicated in AML and CML. HO neutralizes the highly cytotoxic, free-radical producing heme that controls diverse molecular and cellular processes [71]. Of its multiple isoforms, HO-1 is predominantly involved in redox biology and catalyzes the oxidative degradation of cellular heme into free iron, carbon monoxide, and biliverdin, which is immediately converted into bilirubin. HO-1 has shown to exhibit antioxidant, antiapoptotic, and immune-modulating effects by removing pro-oxidant heme and catabolic end-products, thereby providing a cytoprotective and beneficial function to cells. Contradicting evidence shows that HO-1 may promote carcinogenesis and drug-resistance in AML upon induction with chemotherapeutic drugs [72]. Similarly, in CML, HO-1 expression correlates with cell survival, proliferation, and drug resistance [73].

Superoxide dismutase (SOD) catalyzes the conversion of superoxide to molecular oxygen and hydrogen peroxide, further processed into water and molecular oxygen by CAT. In leukemia, however, the activity of SOD remains debatable. Different expression of SOD in leukemia blasts and serum have been noted. For instance, SOD activity was decreased in lymphocytes from ALL [39] and CLL [70] patients, whereas protein expression of SOD, was increased in the serum collected from acute leukemia patients and the decrease in the SOD2 was linked to disease regression [74]. Analyses of SOD in both leukemic blasts and serum from patients could explain the inconsistency in SOD elevation or reduction in leukemia. SOD inhibition has shown to induce apoptosis by the elevation of superoxide and free-radical mediated mitochondrial damage in all four subtypes of leukemia [75].

Catalase (CAT) catalyzes the conversion of hydrogen peroxide into water and molecular oxygen. Overexpression of CAT is cytoprotective to cells, as it increases life span and decreases ROS-induced injury. However, in leukemia, alteration of CAT may lead to increased proliferation, genomic instability, and drug resistance. The increase or decrease in catalase activity has different implications on both myeloid and lymphocytic type leukemias. In AML cell lines and CML samples, an elevation in CAT activity was observed compared to normal granulocytes [76]. Increased CAT activity results in lowered ROS levels, which promote proliferation and resistance to chemotherapeutics [77]. Contrarily, decreased CAT activity results in elevated ROS levels associated with lipid, DNA damage, and genomic instability.

Glutathione (GSH) is the most abundant antioxidant present within the cells and is consumed in the cells to restore other antioxidants and remove OS. Glutathione peroxidase catalyzes the conversion of GSH to oxidized glutathione disulfide (GSSG). When cellular GSH is low, GSSG is reduced back to GSH through a reaction that utilizes NADPH, thereby forming a redox cycle. A depletion in GSH or reduced GSH/GSSH ratio leads to OS, implicated in cancer progression. In contrast, elevated GSH levels increase the antioxidant capacity resulting in resistance to OS in cancer cells. GSH elevation has been observed in hematological malignancies and is associated with chemoresistance. Study shows that elevated GSH levels in leukemic blasts of children and adults with ALL were associated with increased resistance to melphalan, daunorubicin, and prednisolone [78].

Thioredoxin (Trx) system regulates cellular ROS levels and helps maintain redox homeostasis of leukemia cells. The elimination of hydrogen peroxide processed from superoxide anion involves Trx, thioredoxin reductase, and NADPH. Peroxiredoxin reduces hydrogen peroxide, and the oxidized peroxiredoxin is, in turn, reduced by Trx. Furthermore, oxidized Trx is recycled by thioredoxin reductase using NADPH [79]. In the presence of thioredoxin reductase, Trx takes electrons from NADPH, transfers them to the active site of Trx, then uses the electrons to decrease protein disulfides [80]. The Trx system has been implicated in carcinogenesis by promoting cell proliferation, angiogenesis, metastasis, and inhibiting apoptosis signal-regulating kinase 1. In hematological malignancies, upregulation of Trx expression is associated with aggressive disease and shorter relapse interval, as observed in samples from patients with primary and relapsed AML and ALL [49].

## ROS modulated therapy in AML

### The pro-oxidant approach

Leukemia cells are known to generate high ROS levels, and chemotherapy, which is the mainstay treatment in leukemia, is associated with elevated ROS levels. The purine analog, cytarabine, effectively induces remission in AML, although it has high toxicity profiles and a short duration of clinical response with a 5-year event-free survival [81]. Cytarabine induces ROS elevation in both leukemia cells and nonproliferating cells and alters the antioxidant levels in these cells. The early induction of ROS and altered antioxidant expression contribute to OS and eventually lead to apoptotic death [82]. Vincristine, a mitotic inhibitor, has successfully treated ALL when combined with anthracyclines, steroids, or asparaginase, though with early relapse of 15 months. Much like cytarabine, an early elevation in ROS is seen with the initiation of treatment with vincristine [83].

Daunorubicin, idarubicin, and mitoxantrone are anthracyclines which, when in their semiquinone free radical form, can induce direct DNA damage or promote ROS production by interacting with molecular oxygen [84]. Furthermore, anthracyclines complex with free iron in cells, leading to a Fenton reaction in which the combination of iron and hydrogen peroxide results in a further upregulation of ROS. Arsenic trioxide, an anti-leukemic agent that has shown encouraging results in treating relapsed acute promyelocytic leukemia, induces ROS production through Trx inhibition and NOX activation [85]. In a recent study, the pro-oxidant approach of high-dose ascorbate in combination with arsenic trioxide in AML and acute promyelocytic leukemia was evaluated and proved that leukemic cell apoptosis was related to increased ROS and OS [86].

Histone deacetylase inhibitors (HDACi) induce ROS production as part of their cytotoxic mechanism by elevating NOX2 expression and increasing the thioredoxin-binding protein-2 (TBP-2) gene expression and promoting the expression of the Bcl-2 family member Bid. Vorinostat, an HDACi, was reported to induce ROS, DNA damage, and eventually apoptosis in leukemic cell lines [87]. The combination of HDACi with standard chemotherapy agents could potentiate their effectiveness in clinical outcomes [11]. The relation between Proteasome Inhibitors and ROS production is better established in hematological malignancies than in solid tumors. Bortezomib, the first proteosome inhibitor approved by the FDA, has shown promising clinical outcomes in mantle cell lymphoma and myeloma and may have therapeutic benefits in leukemia. In vitro studies have indicated that bortezomib inhibits proliferation in AML blasts and enhances apoptosis [88].

**Fig. 2:**
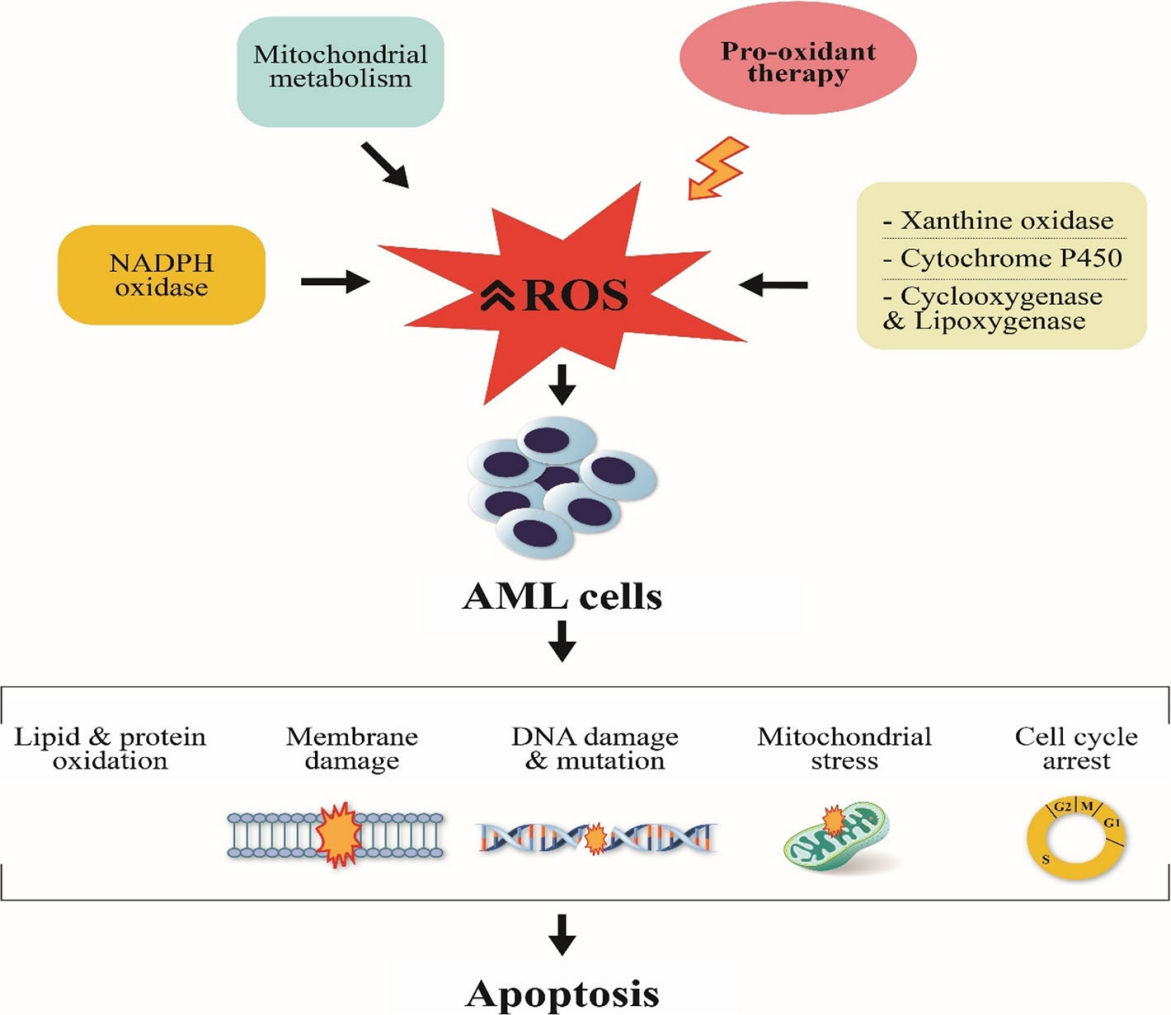
Mechanisms by which the pro-oxidant approach induces cell death in leukemia treatment

The pro-oxidant approach has been preferred over the past years due to the hypothesis that amplification of ROS levels by chemotherapy and other ROS-producing agents induces apoptotic death and leads to tumor regression. Apart from increasing ROS, this approach can lead to damage of lipids, proteins, and DNA, mutations, mitochondrial stress, reduced antioxidant capacity, and cell cycle arrest, all of which have been implicated in cell injury and cell death **(**Fig. 2**)**. However, prolonged exposure to chemotherapy-induced ROS may induce chemoresistance and lead to increased genetic instability in cancer cells due to ROS-induced mutations [56, 89]. In conclusion, chemotherapy-induced ROS may inhibit the antioxidant defense system leading to further upregulation of ROS and eventually apoptotic death of AML cells [90].

### The antioxidant approach

**Fig. 3:**
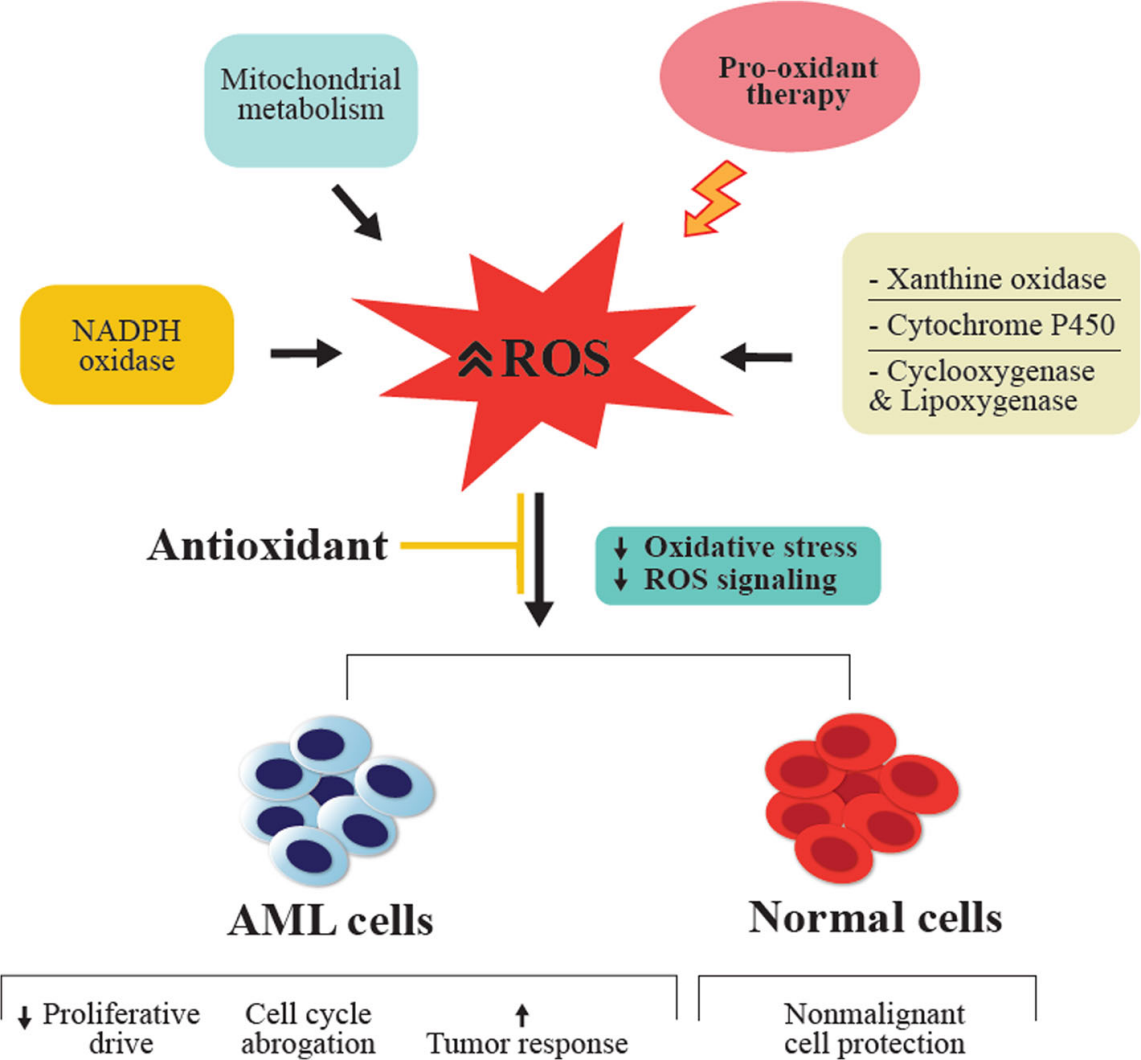
Mechanisms by which antioxidants counteract deleterious effects of pro-oxidant therapy. Antioxidant application to pro-oxidant therapy reduces OS, ROS signaling, and proliferative drive. Furthermore, it induces cell cycle suppression, increases tumor response of AML cells, and protects nonmalignant cells

Antioxidant use to counteract the deleterious effects of ROS or cytotoxicity induced by chemotherapy/radiotherapy in several diseases, including leukemia, was previously frowned upon in clinical practice. Several studies argue that antioxidants may reduce the effectiveness of these agents by protecting not only healthy cells but also malignant cells [91]. To balance the deleterious effects of pro-oxidant therapy, antioxidants reduce ROS signaling, decrease proliferative drive and abrogate the cell cycle, and through these mechanisms, may reduce tumor burden and protect healthy cells from oxidative damage **(**Fig. 3**)** [92]. Nutrient antioxidants which are exogenous sources of antioxidants obtained from food supplements are vitamins A, C, E, carotenoids, flavonoids, trace elements, polyphenols, melatonin, to mention a few, with vitamin C being the most widely used supplement.

Evidence shows that when antioxidants are administered concurrently with chemotherapy, they enhance the cytotoxicity of chemotherapy against malignant cells, protect healthy cells and tissue against treatment-related toxicities, increase patient survival and treatment response, and do not interfere with chemotherapy [93, 94]. In addition to this, supplementation with antioxidants and other nutrients may restore the body’s’ natural antioxidant vitamins and minerals depleted during chemotherapy, which may enhance the patients’ health status. In AML and CML patients, who are reported to have reduced antioxidant capacity, antioxidants have shown to inhibit both the initiation and promotion of carcinogenesis [7]. With antioxidant application in cancer therapy, the challenge is determining which dose intensity to apply: the preventive dose (low dose), which protects both normal and tumor cells or therapeutic dose (high dose), which inhibits the growth of tumor cells but not normal cells.

Studies, both in vitro and in vivo, have shown that combining antioxidants with specific chemotherapeutic agents provides positive benefits and enhances patients’ survival [95]. In a systematic review conducted by Nakayama et al. on the concurrent use of dietary antioxidants with chemotherapy/radiotherapy, it was reported that GSH, vitamin E and N-acetylcysteine (NAC) were the most frequently used in different types of cancers, including leukemia [96], with GSH being the most studied [94]. The effect of antioxidants and a healthy lifestyle of fruit, vegetables, nuts, and seeds were explored to reduce the risk of infectious complications in CLL. Results suggested that antioxidants stimulated immune response and lowered the incidence of infectious complications in CLL patients [97]. Compound kushen injection (CKI), studied as a promising prospect for the treatment of AML, showed the potential of regulating ROS levels to prevent AML relapse as an antioxidant. This study showed that CKI inhibited the proliferation of both hyperleukocytic and non-hyperleukocytic AML cells, and promoted apoptosis of AML cells. Additionally, CKI inhibited intracellular ROS levels by increasing peroxiredoxin 2 and peroxiredoxin 3 expression and decreasing Trx1 expression [98].

The PI, Ixazomib, was evaluated for use in human AML cell lines expressing the mutated nucleophosmin-1 gene. Superoxide induction after ixazomib treatment enhanced apoptosis and cytotoxicity that was reduced by treatment with NAC. The study proved that antioxidants could reduce the toxicity of pro-oxidant drugs [99]. NAC administration elevated antioxidant activity and showed potential in preventing leukemia initiation and reducing DNA damage induced by HL-60 leukemia cells [100]. In children with ALL on chemotherapy, adequate supplementation with Vitamin C and E and β-carotene was associated with a decreased risk of chemotherapy-related toxicity and a lower incidence of infections. In contrast, a lower intake of antioxidants was associated with increased adverse effects [101]. Because several antioxidants stimulate apoptotic pathways and chemotherapy also induces apoptosis by harming DNA, complementary effects between chemotherapeutics and antioxidants exist [94]. The polyphenols, resveratrol, and curcumin exhibit pro-oxidant and antioxidant effects, whether in combination with other natural antioxidants or classical chemotherapy and may be promising future strategies in chemoprevention and chemotherapy of hematological malignancies [102, 103].

In summary, to obtain the desired beneficial results from chemotherapy in AML patients, it may be necessary to supplement treatment with nutritional antioxidants. Targeted nutritional therapies with antioxidants may enhance the patients’ health status, reduce treatment-related toxicities, and increase chemotherapeutic efficiency, thereby improving AML patients’ outcomes.

## Conclusion

AML remains a daunting challenge with high relapse rates and poor patient outcomes despite the current treatment advancements. ROS and OS have been implicated in cellular injury and leukemogenesis. Leukemia cells exhibit elevated ROS levels, of which several identified pathways further increase ROS generation. Alterations in mitochondrial metabolism, cytochrome P450, leukemic oncogenes, expression of metabolic enzymes, and a defective antioxidant capacity play a role in leukemogenesis. The oncogene Jab1/COPS5 is aberrantly expressed in several malignancies, including leukemia. Overexpression of Jab1 and Trx was associated with disease progression and poor prognosis in relapsed AML-M5, thereby presenting a new target in the ROS pathway.

In recent years, pro-oxidant therapy has been preferred for front-line treatment as chemotherapy induces apoptosis and tumor regression through ROS elevation. Antioxidant use to minimize oxidative damage and alleviate chemotherapy-induced toxicity has been frowned upon by clinical oncologists owing to controversies on reduced chemotherapy efficacy with antioxidant use. However, many studies support the use of supplemental antioxidants with chemotherapy to enhance the cytotoxicity of chemotherapy against malignant cells, reduce treatment-related toxicities, boost the patients’ health status, and improve treatment response. Therefore, antioxidant supplements should be administered during chemotherapy in AML patients to improve the efficiency of therapy, treatment outcome, and patient survival.

## Data Availability

Not applicable.

## Abbreviations

AML: Acute myeloid leukemia
ALL: Acute lymphoblastic leukemia
BCR-ABL: Breakpoint cluster region-Abelson
Bcl-2: B-cell lymphoma 2
CAT: Catalase
JAB1/COPS5: C-Jun activation domain-binding protein-1
CML: Chronic myeloid leukemia
CKI: Compound kushen injection
GSH: Glutathione
HSCs: Hematopoietic stem cells
HO: Heme oxygenase
IDH: Isocitrate dehydrogenase
MtETC: Mitochondrial electron transport chain
NAC: N-acetylcysteine
NADPH: Nicotinamide adenine dinucleotide phosphate
NOX: NADPH oxidase
OS: Oxidative stress
ROS: Reactive oxygen species
SOD: Superoxide dismutase
Trx: Thioredoxin
8-OHG: 8-oxoGuanine
